# Significance of admission temperature and impact on mortality in critically ill neurological patients

**DOI:** 10.1186/cc9740

**Published:** 2011-03-11

**Authors:** F Rincon, C Schorr, C Hunter, B Milcareck, R Dellinger, J Parrillo, S Zanotti

**Affiliations:** 1 Thomas Jefferson University, Philadelphia, PA, USA; 2 Cooper University Hospital, Camden, NJ, USA

## Introduction

The purpose of this study is to test the hypothesis that hyperthermia is associated with increased mortality after neurological injury using a robust multicenter ICU database.

## Methods

A multicenter cohort study using the Project IMPACT critical care database of ICUs at 120 US hospitals between 2003 and 2008. Patient inclusion criteria were age older than 17 years, acute neurological injury within 24 hours of admission (acute ischemic stroke (AIS), subarachnoid hemorrhage (SAH), intracerebral hemorrhage (ICH), subdural hematoma (SDH), and traumatic brain injury (TBI)), and admission to the ICU. Patients were divided into three main groups based on definitions of hyperthermia and hypothermia in the ICU. Hyperthermia was defined as temperature greater than 37.5°C, hypothermia as a temperature lower than 36.5°C, and normothermia, not classified as hyperthermia or hypothermia. The outcome measure was in-hospital mortality.

## Results

Over the 8-year period, the Project IMPACT database contained data on more than 700,000 ICU admissions. We found 16,889 patients that met the inclusion criteria. The mean age was 61 ± 19 years, 9,339 (56%) were male, and 12,634 (76%) were white. Of these, 3,081 (18%) had AIS, 2,413 (14%) had SAH, 4,315 (26%) had ICH, 2,748 (16%) had SDH, and 4,317 (26%) had TBI. The mean admission temperature was 37.5 ± 3°C and the overall mortality was 3,628/16,676 (22%). Of the total cohort, 7,878 (47%) had hyperthermia, 689 (4%) had hypothermia, and 8,167 (49%) were normothermic. The hyperthermia group had a high in-hospital mortality (2,180/7,822 (28%)) compared with normothermia (1,169/8,167 (14%)) but the hypothermia group had significantly higher in-hospital mortality (279/687 (41%)). In a preliminary multivariate model controlling for potential confounders (age and gender), hyperthermia (OR, 1.2; 95% CI, 1.1 to 1.23) and hypothermia (OR, 1.9; 95% CI, 1.7 to 2.1) increased the odds of hospital mortality.

## Conclusions

Among critically ill neurological patients admitted to the ICU, hyperthermia and hypothermia are associated with increased in-hospital mortality compared with normothermia. The implications of these findings require further study.
